# Blocking of EphA2 on Endometrial Tumor Cells Reduces Susceptibility to Vδ1 Gamma-Delta T-Cell-Mediated Killing

**DOI:** 10.3389/fimmu.2021.752646

**Published:** 2021-10-07

**Authors:** Robert Hudecek, Barbora Kohlova, Ingrid Siskova, Martin Piskacek, Andrea Knight

**Affiliations:** ^1^ Department of Gynecology and Obstetrics, University Hospital Brno and Masaryk University, Brno, Czechia; ^2^ Faculty of Medicine, Department of Pathological Physiology, Masaryk University, Brno, Czechia

**Keywords:** gamma-delta T cells, endometriosis, peritoneal fluid, tyrosine kinase EphA2, cytotoxicity, innate immunity

## Abstract

**Background:**

Endometriosis is a common gynecological disease characterized by the presence of endometrial tissue outside the uterus causing chronic inflammation, severe pain, and infertility. However, the innate immunity of gamma-delta (γδ) T lymphocytes in endometriosis has not been characterized. Women with endometriosis present numerous endocrine and immune dysfunctions and elevated risk for endometrial, ovarian, and breast cancers. The tyrosine kinase EphA2 is often overexpressed in cancer including endometrial carcinoma.

**Methods:**

We analyzed Vδ1 and Vδ2 γδ T cells in peripheral blood and paired peritoneal fluid samples in endometriosis patients (*n* = 19) and compared the counts with that of age- and sex-matched healthy donors (*n* = 33) using flow cytometry. Vδ1 and Vδ2 T cells isolated from healthy donors were used against KLE, RL-95, and Ishikawa endometrial tumor cells in 4 h flow cytometric cytotoxicity assays. The EphA2 blocking studies were performed using antibody, small-molecule inhibitor ALW-II-41-27, and the CRISPR/Cas9.

**Results:**

We determined Vδ1 T cells substantially reduced in patients’ peripheral blood (*p* < 0.01) and peritoneal fluid (*p* < 0.001). No differences were found for circulating Vδ2 T cells compared with peritoneal fluid samples. We observed inherent cytotoxic reactivity of Vδ1 and Vδ2 γδ T lymphocytes against endometrial tumor cells. Importantly, we found reduced specific lysis of EphA2-positive cell lines KLE and RL-95 by Vδ1 T cells in the EphA2 antibody blocking studies and by the EphA2 inhibitor. Furthermore, Vδ1 T-cell-mediated killing was significantly decreased in RL-95 cell EPHA2 knockout. Finally, potent cytolytic activity exerted by Vδ1 T cells was significantly reduced in EPHA2 knockouts in renal A-498 and colon HT-29 carcinoma cell lines.

**Conclusions:**

We determined variable levels of Vδ1 and Vδ2 γδ T cells in endometriosis patients. We observed inherent cytotoxic reactivity of γδ T-cell subsets against endometrial cell lines. Specifically, we found that blocking of EphA2 expression resulted in significant inhibition of endometrial tumor killing mediated by Vδ1 γδ T cells. These results suggest that EphA2 is involved in tumor cell lysis and contributes to susceptibility to Vδ1 γδ T cells cytotoxic reactivity.

## Introduction

Endometriosis is a hormone-dependent gynecological disease characterized by the presence of endometrial tissue outside the uterine cavity. The disease affects around 10% of reproductive-aged women (1). Retrograde menstruation is accepted for the pathogenesis when menstrual endometrial tissue fragments and viable cells escape apoptosis, evade normal immune surveillance, enter into peritoneal cavity where adhered, develop a blood supply, and grow into endometriosis lesions (1, 2). Hormonal treatments are believed to reduce proliferation of endometrial lesions by reducing estrogen activity. Increased concentrations of prostaglandins have been reported in peritoneal fluid of endometriosis patients and may be involved in the progression of the disease (3). It is well established that women with endometriosis exhibit numerous endocrine and immune dysfunctions. Specifically, they display aberrant numbers of immune cells and cytokines present in the plasma and peritoneal fluid (PF), which has been shown to contribute to chronic pain and infertility described by endometriosis women (4–7). The immune cells including macrophages, natural killer (NK) cells, cytotoxic T cells, and dendritic cells that lost the ability to effectively detect and destroy autologous endometrial menstrual tissue contribute significantly to the development of acute and chronic inflammation. In addition to decreased NK cell cytotoxicity (8–12) enhanced activation of monocytes and peritoneal macrophages (13, 14) have been well documented. It is still uncertain whether the aberrant activity of these immune cells causes endometriosis or whether they act as secondary enhancers of the disease. Recent evidence suggests that biology of endometriosis significantly overlaps those considered to be hallmarks of cancer and essential alterations in cell physiology including sustained proliferative signaling, evasion of growth suppressors, activation of invasion and metastasis, induction of angiogenesis, resistance to cell death, compromised immune detection, tumor promoting inflammation, and genome instability (15). It is understood that women with endometriosis present elevated risk for cancer by 90% for ovarian cancer, 40% for non-Hodgkin’s lymphoma, and 30% for breast cancer. Many women with endometriosis are also diagnosed with polycystic ovary syndrome (PCOS).

Endometrial cancer (EC) is the most common malignancy of the female reproductive system (16). It tends to develop after menopause in women with a median age at onset of 63 years. Several risk factors have been identified, such as obesity (17), diabetes, PCOS, and infertility. Endometrial carcinoma arises from the lining of the uterus and can be broadly divided into two types: endometrioid carcinomas, affecting approximately 80% of patients, which can be graded according to the relative proportion of solid tumor and the nonendometrial carcinomas, which have a hormone-independent pathogenesis and unknown precursor lesion (16). An early stage EC patients’ prognosis is generally favorable.

Human gamma-delta (γδ) T lymphocytes play critical roles in immune surveillance mediating potent inflammatory response and contributing to prominent tumor killing (18, 19). γδ T cells account for 1%–10% of T cells in the peripheral blood in adults and are often enriched as resident cells within the solid organs and mucosal tissues. They are considered the first line of innate immune defense, but they also have the possibility to create immunological memory and therefore also belong to adaptive immunity (20, 21). In contrast to conventional αβ T cells, γδ T cells display a non-MHC-restricted antigen recognition. Human γδ T cells can be divided according to their T-cell receptor (TCR) delta chain usage into two major populations, namely Vδ1 and Vδ2 T cells (22). Recent study highlighted the role of γδ T cells in cancer as the most significant favorable prognostic immune subset associated with overall survival outcomes across 39 malignancies (23). However, to our knowledge, γδ T cells in endometriosis patients have not been characterized.

The Eph receptors represent the largest family of receptor tyrosine kinases. Together with their respective ligands, they have been extensively studied for the roles they play during embryonic development, particularly within the central nervous system (24). As a unique feature, bidirectional signaling in Eph/ephrin ligands between cells is fundamentally involved in developmental processes, such as axonal guidance, remodeling of blood vessels or correct formation of crypt and villi in the intestinal epithelium (24, 25). Some Eph receptors, especially EphA2 is often overexpressed and functionally altered in many cancers including breast (26), ovarian (27), and endometrial (28, 29) carcinomas, which correlated with, e.g., increased invasiveness, increased metastatic potential, prominent vascularization, and consequently with poor patient outcome. Most recently, EphA2 has been identified as a stress antigen recognized by a Vδ1 TCR (30).

We conducted the present study to determine the numbers of γδ T-cell subsets in endometriosis patients. We demonstrate for the first time the prominent cytotoxicity of γδ T cells against endometrial tumor cell lines. Next, we show that the EphA2 receptor is highly important in tumor recognition and killing by Vδ1 γδ T cells.

## Materials and Methods

### Patient Characteristics

Patients (*n* = 19) have been enrolled from the Department of Gynecology and Obstetrics, Faculty Hospital Brno. The study was approved by the local institutional ethics committee of the Faculty of Medicine, Masaryk University. The study was performed in accordance with the Declaration of Helsinki. Written informed consents were obtained from all patients. Endometriosis was assessed according to the revised American Fertility Society (r-AFS) classification during laparoscopy. Patients received no hormonal therapy for a minimum of 3 months prior to laparoscopic surgery.

The patient characteristics are shown in **Table 1**.

**Table 1:** Study subjects.

Parameters	Endometriosis patients	Controls	
Number (*n*)	19	33	
Age (years)			
Median	33	29	
Range	24–48	18–48	
Disease stage*			
I	3		
II	4		
III	5		
IV	7		
Menstrual cycle			
EPP	8	
LPP	3	
ESP	5	
LSP	3	

*Classification according to r-AFS.

EPP, early proliferation phase; LPP, late proliferation phase; ESP, early secretory phase; LSP, late secretory phase.

### Sample Collection and Preparation

Peripheral blood (PB) and peritoneal fluid (PF) samples were obtained from endometriosis patients and were processed within 2 h of collection. PF samples were taken during the planned surgery from lower pelvis cavity by fine needle suction from cavum Douglasi at the opening phase of diagnostic laparoscopy prior the surgical procedure as less invasive technique for more patients than tissue biopsy. At the same time, it allows to obtain sufficient volumes of biological material for subsequent analysis. Buffy coats from age- and sex-matched healthy volunteers (*n* = 33) were collected at the Transfusion and Tissue Bank, Faculty Hospital Brno. Peripheral blood mononuclear cells (PBMCs) were isolated from blood by density gradient centrifugation using Lymphoprep (Stem Cell Technologies) following the manufacturer’s recommendations. Plasma samples were collected and stored at −80°C.

### Cell Culture

Endometrial carcinoma cell lines KLE (ATCC^®^ CRL1622™) and RL95-2 (ATCC^®^ CRL1671™) were purchased from ATCC (American Type Culture Collection, VA, USA). Ishikawa cell line was purchased from Sigma-Aldrich (St. Louis, MO, USA). KLE and RL95-2 cell lines were maintained in Dulbecco’s modified Eagle’s medium (DMEM/F12) supplemented with 10% fetal bovine serum (FBS) and 2% penicillin/streptomycin (all Thermo Fisher Scientific); RL95-2 cells with the addition of 5ug/ml of insulin (Sigma-Aldrich, MO, USA). The Ishikawa cell line was maintained in MEM supplemented with 5% FBS and 2% penicillin/streptomycin.

In addition, human tumor cell lines including myeloma (U266, EJM) and chronic myeloid leukemia (LAMA-84, KYO-1) were purchased from DSMZ (German Collection of Microorganisms and Cell Cultures GmbH, Germany). Renal (A-498), prostate (DU-145), breast adenocarcinoma (MCF-7) and histocytic lymphoma (U937), chronic myeloid leukemia (K562), acute monocytic leukemia (THP-1) and glioblastoma (U87 MG) cells were purchased from ATCC. Glioblastoma cells (U-373 MG and U251 MG) were purchased from The European Collection of Authenticated Cell Culture (ECACC). Myeloma cell lines (OPM-2, LP-1, KMS-11) were a kind gift from Dr. Krejci (Institute of Biology, Masaryk University Brno). Cells lines (U266, LAMA-84, KYO-1, K562, U-937, THP-1, EJM) were cultured in RPMI-1640 containing 10% FBS, 2mM L-glutamine, and 2% penicillin/streptomycin. Cell lines (A-498, DU-145, HT-29, U-87 MG, MCF-7) were cultured in modified Eagle’s medium (MEM) (Sigma Aldrich) with 10% FBS, 2 mM l-glutamine, and 2% penicillin/streptomycin. The MCF-7 cells were supplemented with nonessential amino acids (NEAA, Sigma Aldrich). Cell lines (U251 MG, U-373 MG) were cultured in DMEM/F12 with 10% FBS. All cells were grown at 37°C in 5% CO_2_ atmosphere up to 70%–80% confluence; adherent cells were harvested by using gentle dissociation solution TrypLE (Gibco, Thermo Fisher Scientific) and counted by using Trypan blue exclusion.

The EphA2 inhibitor ALW-II-41-27 was purchased from MedChem Express (Monmouth Junction, NJ, USA). It was dissolved in sterile DMSO at 10 mM stock concentration and solution stored in aliquots at −20°C.

### Flow Cytometric Phenotyping

The cell phenotype was assessed by fluorescence-activated cell sorting (FACS) by using staining with the monoclonal antibodies MICA, MICB, CD112, CD155, B7-H6 (R&D Systems, clone 875001), and EphA2 (R&D Systems, clone 371805). The tumor cell lines were harvested, washed with cold phosphate-buffered saline (PBS, Sigma) containing 2% FBS, and incubated for 30 min on ice with fluorescently labelled monoclonal antibodies. Gamma-delta T cells were identified in freshly isolated PBMCs labelled with CD3 (Thermo Fisher Scientific, clone SK7), Vδ1 TCR (Thermo Fisher Scientific, clone TS8.2), Vδ2 TCR (BD Pharmingen, clone B6) or Vδ2 TCR (Sony, clone B6). CD27 (BD Pharmingen, clone M-T271), and CD45RA (Exbio, clone MEM-56) were used for immunophenotyping. Samples were washed and acquired using FACSCanto^®^ (BD Biosciences) and data analyzed using FlowJo^®^ software (FlowJo, Ashland, OR, USA). Forward and side scatter gating were used to discriminate live cells from dead cells and γδ T cells were derived from SSC *vs.* FSC-gated bulk PBMCs with doublet exclusion (FSC-A *vs.* FCS-H). To determine the placement of the gates, appropriate fluorescence minus one (FMO) and unstained controls were used.

### Isolation of Polyclonal Vδ1 and Vδ2 γδ T Lymphocytes

Fresh γδ T-cell populations were sorted by positive selection using anti-TCR Vδ1 (Beckman Coulter, clone R9.12) or anti-TCR Vδ2 (BD Pharmingen, clone B6) monoclonal antibodies and magnetic microbeads (Miltenyi Biotec, Germany) according to manufacturer’s instruction. The cell purity was routinely greater at 97%.

### Cytotoxicity Assay

Freshly sorted Vδ1 or Vδ2 γδ T lymphocytes were incubated with tumor target cells at indicated effector:target (E:T) 5:1 and 10:1 ratios in duplicates for 4 h co-culture at 37°C as described previously (31). Briefly, tumor target cells were washed in Hank’s buffered saline solution (HBSS, Invitrogen Life Technologies) to remove FBS and culture media. Cells were resuspended in diluent C (Sigma) and labeled with PKH67 fluorescent dye (Sigma). To-Pro-3 iodide (1 μM in PBS) (Invitrogen Life Technologies) was added immediately prior to the acquisition on the flow cytometer. At least 10,000 target cells were acquired after gating out the green fluorescence of PKH67 dye and the proportion of To-Pro-3 iodide positive cells. Background target cell death was determined from the cells incubated in the absence of effector cells. In the blocking experiments, the EphA2 antibody (R&D Systems, clone 371805) and ALW-II-41-27 EphA2 inhibitor (10 µM, 1 µM) or DMSO as a control were added to tumor cultures prior the cytotoxicity assays.

### RNA Extraction, cDNA Synthesis, Real-Time PCR

Total RNA has been extracted from tumor cell lines using RNeasy Mini kit (Qiagen) according to manufacturer’s instruction. RNA was eluted in RNAse-free water and stored in −80°C. Complementary DNA (cDNA) has been synthesized using 20 ng/µl total RNA that has been reverse transcribed using High-Capacity cDNA Reverse Transcription Kit (Applied Biosystems). The glyceraldehyde 3-phosphate dehydrogenase (GAPDH) housekeeping gene has been used as an internal control by quantitative real-time polymerase chain reaction (real-time qPCR). cDNAs were amplified using TaqMan^®^ Gene Expression Assay (ID : Hs01072272_ml, Applied Biosystems). Samples were analyzed on StepOne™ Real-Time PCR Systems (Applied Biosystems).

### Generation of EPHA2 Knockout by the CRISPR/Cas9 method

The EPHA2 gene knockout was performed with the EPHA2 CRISPR gRNA + Cas9 in Lenti-particles (supplied from antibodies-online GmbH) and used closely following manufacturer’s instructions. Briefly, vector pLenti-U6-sgRNA-SFFV-Cas9-2A-Puro (product number ABIN5252263) was used to generate EPHA2 knockouts in human endometrial cancer line RL95-2, renal carcinoma cell line A-498, and colon carcinoma cell line HT-29. After infection, positive clones were selected by 3.5 μg/ml puromycin, and the single clones were transferred separately into 48-well plates and further passaged. The EPHA2 knockouts were confirmed by flow cytometry after antibody staining (anti-EPHA2, R&D Systems, clone 371805).

### Statistical Analysis

Data analyses were performed using GraphPad Prism5 software (GraphPad Software Inc., La Jolla, CA). The Student’s *t*-test was used to determine significant differences between groups. Differences between sample groups were evaluated with the nonparametric Mann-Whitney *U* test. *p* < 0.05 values were considered to be significant. Data are expressed as mean ± standard deviation (SD).

## Results

### γδ T Cell Subsets in Peripheral Blood and Peritoneal Fluid Samples in Patients With Endometriosis

**Figure 1:**
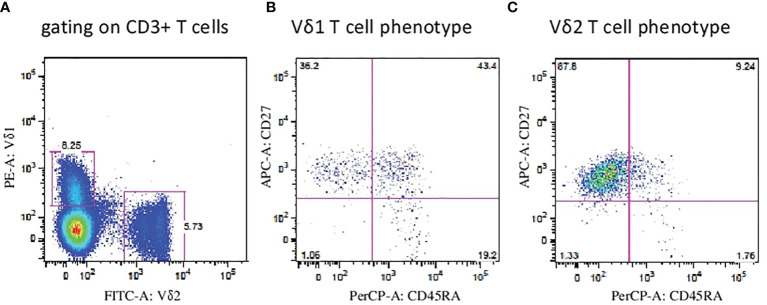
Flow cytometric analysis of Vδ1 and Vδ2 γδ T cells. **(A)** Peripheral blood mononuclear cells (PBMCs) were analyzed where proportion of Vδ1 and Vδ2 γδ T cells among leukocyte gate followed by the percentage of CD3 lymphocytes. Immunophenotyping of Vδ1 **(B)** and Vδ2 **(C)** T cells using the CD27 and CD45RA antibodies to determine the naïve, memory, effector and TEMRA phenotypes was analyzed and representative flow plots are shown.

**Figure 2:**
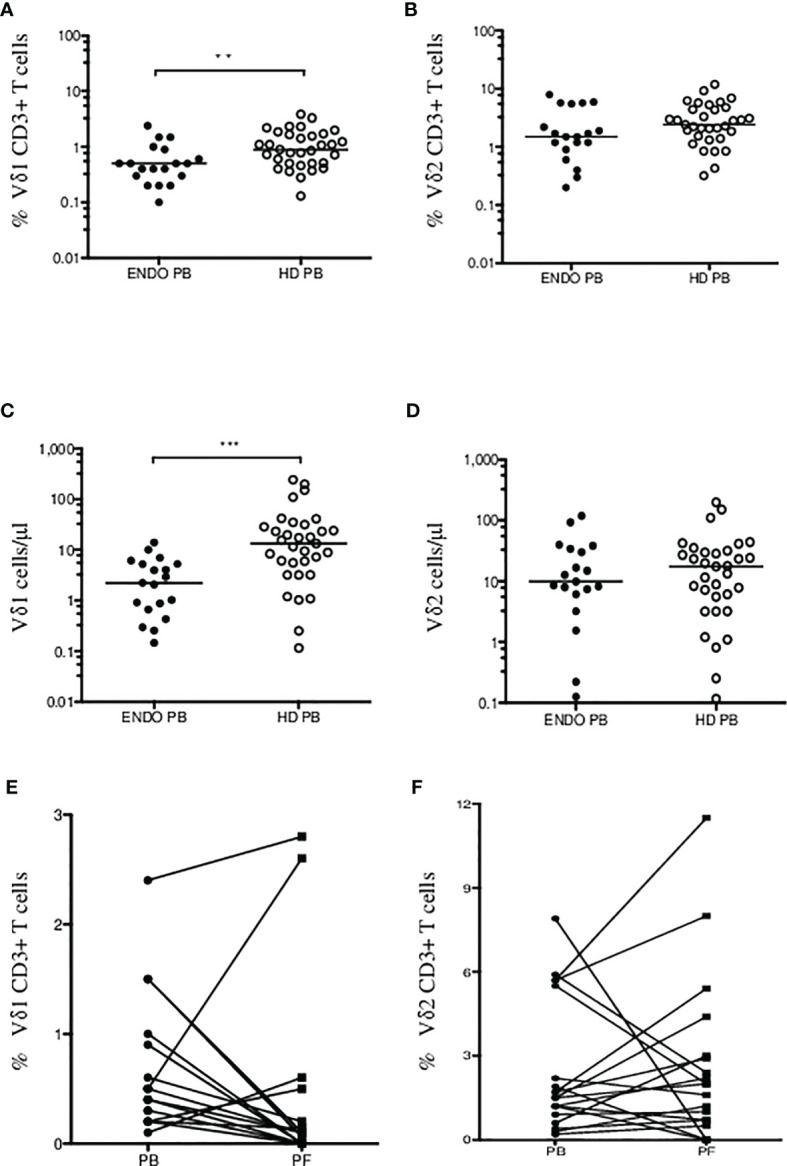
Summary of Vδ1 and Vδ2 γδ T cells in endometriosis patients. Percentage of CD3+ Vδ1 **(A)** and Vδ2 **(B)** γδ T cells in endometriosis patients (ENDO, filled circles) and age- and sex-matched healthy donors (HD, empty circles) are shown. Absolute counts of Vδ1 **(C)** and Vδ2 **(D)** γδ T cells in PB in patients compared with HD were determined. Analysis of Vδ1 **(E)** and Vδ2 T cells **(F)** in patient’s peripheral blood (PB) and paired peritoneal fluid (PF) samples. The median values are shown. Statistically significant differences are presented as **p = 0.008; ***p = 0.0002.

First, we aimed to determine the two major populations of γδ T cells (Vδ1 and Vδ2 subsets, respectively) in peripheral blood (PB) samples from patients with endometriosis (*n* = 19) and compared the frequencies with age- and sex-matched healthy donors (HD, *n* = 33). Flow cytometric analysis of peripheral blood mononuclear cells (PBMCs) where proportion of Vδ1 and Vδ2 γδ T cells among leukocyte gate followed by the percentage of CD3 lymphocytes is shown in **Figure 1A**. Immunophenotyping of Vδ1 (**Figure 1B**) and Vδ2 (**Figure 1C**) using the CD27 and CD45RA antibodies to determine the naïve/memory/effector memory and TEMRA phenotypes was analyzed, and representative flow plots are shown. We found significantly low percentages of Vδ1 T cells in PB (*p* = 0.008) (median 0.5%, range 0.1%–2.4%) in endometriosis patients compared with HD (median 0.9%, range 0.1%–3.8%), as shown in **Figure 2A**, whereas Vδ2 T cells showed no difference between the endometriosis patients (median 1.5%, range 0.2%–7.9%) and healthy controls (median 2.4%, range 0.3%–11.9%), as shown in **Figure 2B**. Next, the absolute counts of Vδ1 and Vδ2 γδ T cells in PB in patients compared with HD were determined in **Figures 2C, D**, respectively. We found dramatically reduced Vδ1 T-cell absolute counts in patients PB (*p* = 0.0002) (median 2.23 cells/µl, range 0.14–14.01) and HD controls (median 13.3 cells/µl, range 0.11–242.1). No differences in Vδ2 T-cell counts were observed between the patients PB (median 9.8 cells/µl, range 0.12–118.7) and HD controls (median 17.3 cells/µl, range 0.11–198).

Second, we analyzed γδ T-cell infiltration in patient’ peritoneal fluid (PF) samples and compared the counts with paired PB. We found most patients with dramatically reduced Vδ1 T cells in PF (median 0.1%, range 0%–2.8%) compared with PB samples (median 0.5%, range 0.1–2.4%) (*p* = 0.001) in **Figure 2E**. Similarly, no significant differences were identified for circulating Vδ2 T cells (median 1.5%, range 0.2%–7.9%) compared with PF samples (median 2.0%, range 0%–11.5%), in **Figure 2F**. These results show for the first time the presence of Vδ1 and Vδ2 γδ T cells in peritoneal fluid in patients with endometriosis.

**Figure 3:**
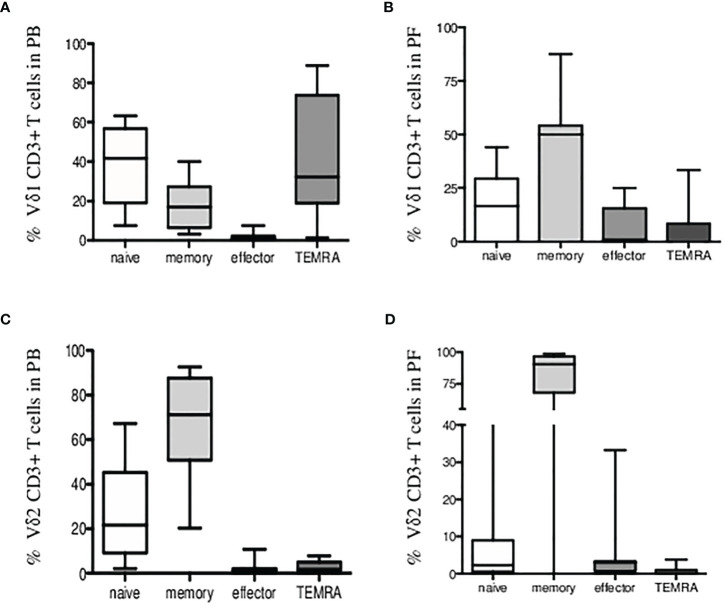
Immunophenotyping of Vδ1 and Vδ2 γδ T cells in endometriosis patients. Percentage of CD3+ Vδ1 T cells **(A)** in peripheral blood and **(B)** in peritoneal fluid samples showing expression of CD27 and CD45RA markers for naïve/memory/effector and TEMRA phenotypes. **(C)** Percentage of CD3+ Vδ2 T cells in peripheral blood and **(D)** in peritoneal fluid samples is shown.

Third, we found most Vδ1 T cells of naïve (CD27+CD45RA+) and TEMRA (CD27-CD45RA+) phenotype in patients PB shown in **Figure 3A**. Peritoneal fluid samples showed majority of Vδ1 T cells at the memory stage of differentiation (CD27+CD45RA-) in **Figure 3B**. Vδ2 T cells in patients PB and PF samples were predominantly of memory phenotypes in **Figures 3C, D**.

### γδ T-Cell-Mediated Killing of Endometrial Tumor Targets

**Figure 4:**
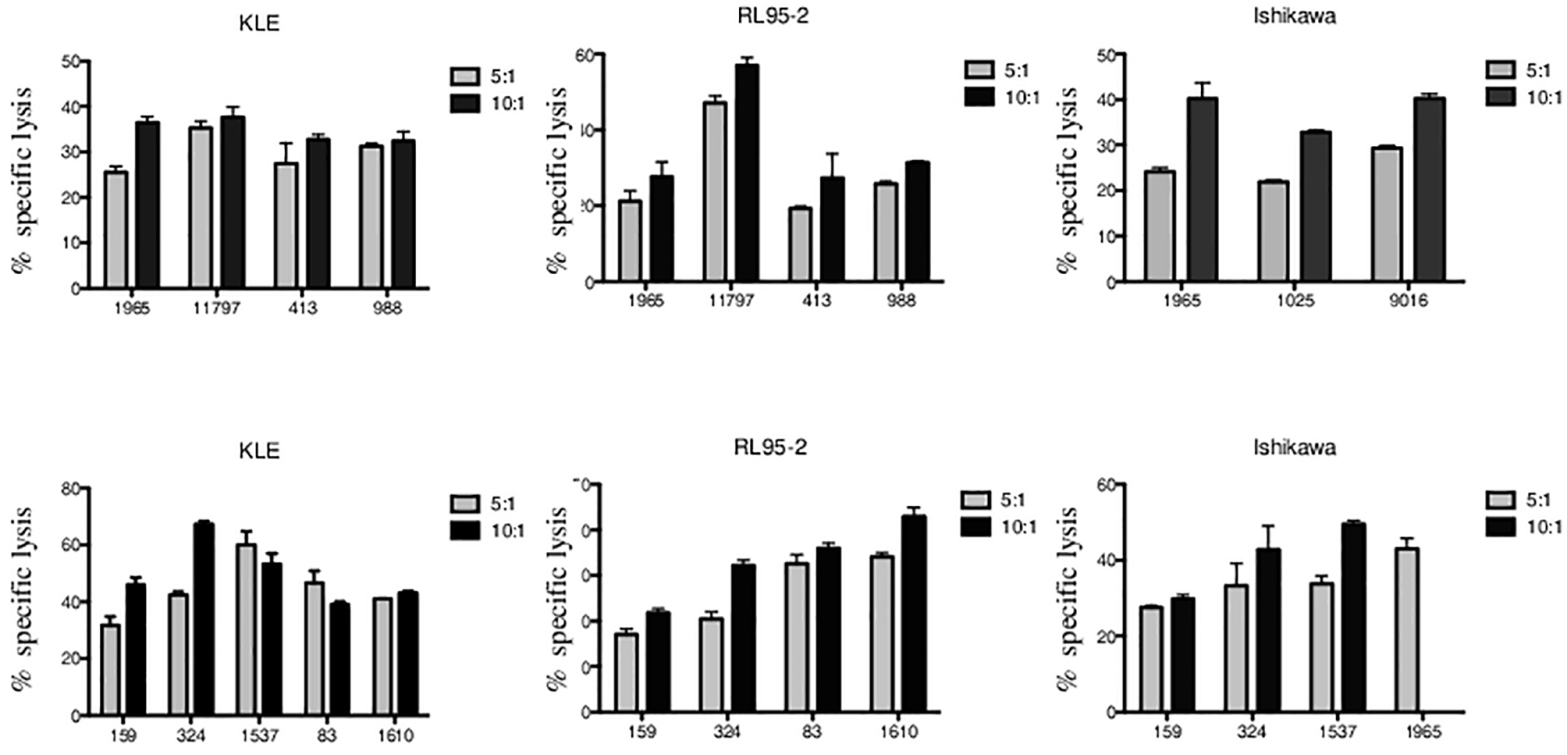
Vδ1 and Vδ2 γδ T-cell-mediated killing of endometrial tumor cell lines KLE, RL95-2, and Ishikawa. Freshly sorted γδ T cells from three to five healthy donors (numbered anonymously) were co-cultured with tumor targets for 4 h, and specific lysis was determined at 5:1 and 10:1 E:T ratio. The results from independent experiments of Vδ1 γδ T-cell cytotoxic reactivity against KLE, RL-95, and Ishikawa is shown as the mean ± SD of sample duplicates. Summary data of specific lysis and prominent cytotoxicity of Vδ2 γδ T cells against KLE, RL-95, and Ishikawa is shown as the mean ± SD of independent experiments performed in duplicates.

We analyzed the cytotoxic function of Vδ1 and Vδ2 γδ T cells freshly sorted from healthy donors against endometrial tumor cell lines including Ishikawa, KLE, and RL95-2. We determined the 4-h killing reactivity shown as percentages of specific lysis of Vδ1 and Vδ2 γδ T cells at 5:1 and 10:1 E:T ratio. All of the tested γδ T lymphocytes efficiently killed the tumor targets. First, the summary of Vδ1 T cell-mediated killing (*n* = 4) at 5:1 E:T against KLE (mean 29.8%, SD 4.2%), RL95-2 (mean 28.4%, SD 12.7%) and Ishikawa (*n* = 3, mean 25.1%, SD 3.8%) in **Figure 4** is shown. Importantly, significant antiendometrial reactivity of Vδ1 T cells was detected at 10:1 E:T against KLE (mean 34.8%, SD 1.3%), RL95-2 (35.9%, SD 14.2%), and Ishikawa (mean 37.8%, SD 4.3%).

Second, summary of Vδ2 T-cell-mediated killing against KLE, RL95-2, and Ishikawa (**Figure 4**) is shown. At low E:T ratio of 5:1, the specific lysis was detected against KLE (*n* = 5, mean 44.4%, SD 10.3%), RL95-2 (*n* = 4, mean 26.0%, SD 8.6%), and Ishikawa (*n* = 4, mean 34.4%, SD 6.4%). Prominent ability of Vδ2 γδ T cells to recognize and kill endometrial tumor targets was observed at 10:1 E:T against KLE (mean 49.8%, SD 11.1%), RL95-2 (mean 33.2%, SD 8.8%) and Ishikawa (mean 40.7%, SD 10%). Altogether, the endometrial tumor killing was comparable for γδ T-cell subsets isolated from different donors and was reproducible between the assays for all cell lines.

### The EphA2 Expressed on Endometrial Tumor Cells Is Involved in Vδ1 T-Cell-Mediated Killing

**Figure 5:**
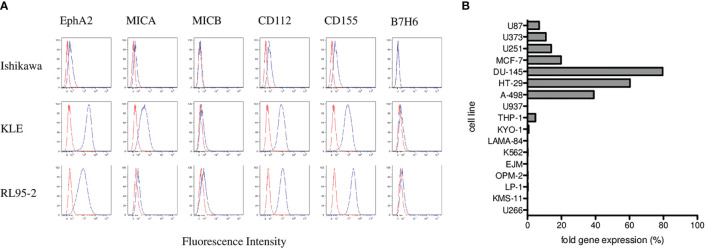
Phenotyping and surface expression of EphA2, MICA, MICB, CD112, CD155, and B7-H6 on target endometrial tumor cell lines including Ishikawa, KLE, and RL95-2 by flow cytometry. **(A)** Representative plots are shown as histograms of the unstained controls (red) and histograms representing the stained samples (blue). Data are expressed as mean fluorescence intensity (MFI, *x*-axis) *versus* number of cells (*y*-axis). **(B)** Summary of the EphA2 expression analyzed by the real-time qPCR in a panel of tumor cell lines presented as fold gene expression.

To elucidate possible mechanisms involved in γδ T cell cytotoxicity, we evaluated several molecules typically involved in γδ T-cell killing including the MICA and MICB as ligands for the NKG2D receptor; CD112 and CD155 as ligands for the DNAM-1 receptor; and ligand B7-H6 for the NKp30 receptor. We analyzed the surface expression of MICA, MICB, CD112, CD155, and B7-H6 on target endometrial tumor cell lines including Ishikawa, KLE and RL95-2 by flow cytometry. Variable expression of these markers is shown in **Figure 5A**. Furthermore, we analyzed the expression of the EphA2 receptor, which is known to be overexpressed in many human malignancies, including endometrial carcinoma. We showed activation and high expression of the EphA2 receptor on KLE and RL95-2 endometrial tumor cell lines but only weak expression on Ishikawa cells in **Figure 5A**. In addition, we determined the EphA2 RNA expression by the real-time qPCR in a panel of tumor cell lines as fold gene expression in **Figure 5B**. We found the highest EphA2 expression in solid tumors including prostate (DU-145), colon (HT-29), and renal (A-498) carcinoma cell lines in contrast to mostly negative hematological cell lines.

**Figure 6:**
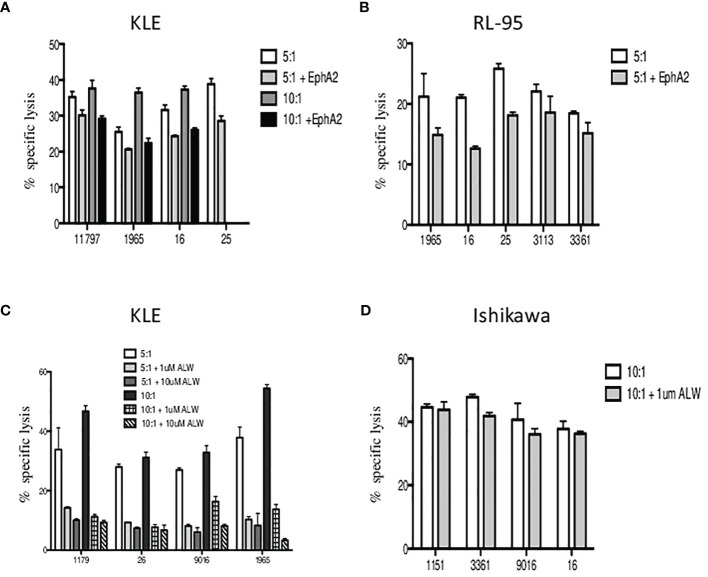
The inhibition of Vδ1 T-cell-mediated killing by blocking of EphA2 expression on endometrial tumor cells. The target cells were preincubated with the EphA2 antibody prior to 4 h cytotoxicity assays and the specific lysis of KLE **(A)** and RL95-2 **(B)** cells was determined at 5:1 and 10:1 E:T ratios. Summary data of specific lysis and cytotoxicity reduction in the presence of EphA2 antibody is shown as the mean ± SD of independent experiments performed in duplicates (HD numbered anonymously). **(C)** Analysis of the *in vitro* effects of the small-molecule inhibitor ALW-II-41-27 at 1 and 10 µM on KLE endometrial tumor cells. Dose-dependent inhibition of cytotoxicity by Vδ1 T cells is shown at 5:1 (pale grey bars) and 10:1 (dark grey bars) E:T ratios. **(D)** Vδ1 T-cell-mediated killing of EphA2-negative Ishikawa endometrial cell line was analyzed at 10:1 E:T with/without the presence of small-molecule inhibitor ALW-II-41-27 (1 µM) and is shown as the mean ± SD of independent experiments performed in duplicates.

Next, we aimed to determine whether the EphA2 is involved in γδ T-cell killing. In the blocking experiments, we first preincubated the target cells with the EphA2 antibody prior to 4-h cytotoxicity assays and then analyzed the specific lysis of KLE (**Figure 6A**) and RL95-2 (**Figure 6B**) target cells in the presence/absence of the EphA2. Interestingly, all Vδ1 T cells isolated from healthy donors killed efficiently both tumor cell lines at 5:1 and 10:1 E:T ratios; however, the cytotoxicity was reduced when the EphA2 receptor was blocked. The inhibitory effect of anti-EphA2 on tumor cell killing was determined for KLE cells in the range 14%–40% (median 25%) and for RL95 cells in the range of 15%–40% (median 26%). Together, these results suggested that EphA2 was recognized by cytotoxic Vδ1 T cells in the tumor killing.

To validate these findings, we then tested the *in vitro* effects of the EPHA2 small-molecule inhibitor ALW-II-41-27 on KLE endometrial tumor cells. We incubated the KLE target cells with ALW-II-41-27 inhibitor at 1 and 10 µM concentrations and showed the specific lysis significantly reduced at both 5:1 and 10:1 E:T ratios in the range 50%–80% (median 71%) (**Figure 6C**). Importantly, the inhibition of cytotoxicity by Vδ1 T cells was shown as dose dependent. Next, we used the EphA2-negative Ishikawa cell line and determined the specific lysis at 10:1 E:T in the presence of ALW-II-41-27 (1 µM). No significant reduction of tumor killing was observed (**Figure 6D**). Of note, the pharmacological effect of inhibitor ALW-II-41-27 on cell viability in drug treatment sample relative to a DMSO control group was determined independently prior the killing assays and no increase of the spontaneous cell lysis was detected after 4h (data not shown). In addition, the inhibitor ALW-II-41-27 showed no change in the surface expression of EphA2, B7-H6 and stress ligands in 4-h cytotoxicity incubation (data not shown).

**Figure 7:**
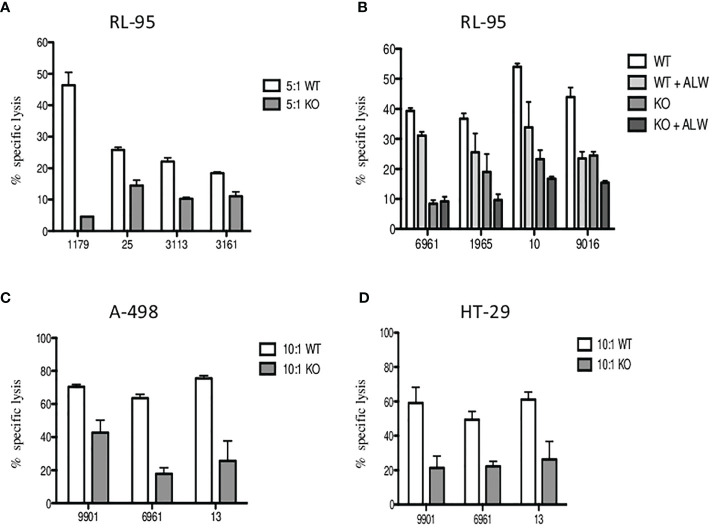
The inhibition of specific lysis of EphA2-positive RL-95 endometrial cell line. **(A)** Freshly sorted Vδ1 T cells were co-cultured with tumor targets for 4 h, and cytotoxicity was determined at 5:1 E:T ratio for the wild type (WT, white bars) and the EPHA2 knockout (KO, grey bars) shown as the mean ± SD of independent experiments performed in duplicates (HD numbered anonymously). **(B)** Similarly, inhibition of specific lysis by Vδ1 T cells was determined at 10:1 E:T ratio for the wild type (WT, white bars) and the EPHA2 knockout (KO, dark grey bars) with/without the presence of small-molecule inhibitor ALW-II-41-27 (1uM) and is shown as the mean ± SD of independent experiments performed in duplicates. **(C)** The EPHA2 knockouts of renal tumor cell line A-498 (grey bars) and colon adenocarcinoma tumor cell line HT-29 **(D)** were generated and specific lysis was compared with WT cells (white bars) at 10:1 ratio. Significant inhibition of cell lysis mediated by tumor-reactive Vδ1 T cells was shown as the mean ± SD of independent experiments performed in duplicates.

Finally, to confirm the inhibition of the EPHA2 resulting in significantly decreased *in vitro* tumor cell death, we generated EPHA2 knockouts (KO) in endometrial cell line RL95-2 by the CRISPR/Cas9 method. The loss of EPHA2 significantly reduced specific lysis by 35%–90% (median 45%) by Vδ1 T cells in knockout *versus* wild type (WT) at 5:1 E:T ratio is shown in **Figure 7A**. Next, we observed the Vδ1 T-cell cytotoxicity inhibition of RL95-2 WT *versus* KO cells and also in the addition of ALW-II-41-27 inhibitor (1 µM) at 10:1 E:T in **Figure 7B**.

To further verify these results, we generated EPHA2 knockouts in A-498 (renal) and HT-29 (colon) tumor cell lines which had previously showed the highest EphA2 expression in **Figure 5B**. The summary of Vδ1 T cell cytotoxicity results is shown for A-498 in **Figure 7C** and HT-29 in **Figure 7D**. The A-498 KO cells showed significant protection from specific lysis mediated by Vδ1 T cells compared with WT cells at 10:1 ratio between 40% and 77% (median 61%). Similarly, HT-29 KO cells presented significant reduction of tumor killing than WT cells at 10:1 ratio between 42% and 75% (median 44%).

In summary, we evaluated Vδ1 γδ T-cell cytotoxicity against tumor cells and found consistently that EphA2 expressed on cancer cells show susceptibility to cell lysis by tumor-reactive Vδ1 T cells.

## Discussion

It is well accepted that women with endometriosis exhibit numerous immune dysfunctions and that the immune system plays a central role in its etiology, infertility, increased risk of ovarian carcinoma, or poor pregnancy outcomes (32). Pathogenesis of endometriosis is poorly understood, and the incomplete phenotyping of immune cells within the endometrium and peritoneal fluid of women with the disease warrants urgent research to identify biomarkers that could be used to predict or verify the disease.

In this study, we determined for the first time the numbers of Vδ1 and Vδ2 γδ T-cell subsets in peripheral blood and peritoneal fluids in patients with endometriosis. We observed dramatically reduced numbers of circulating Vδ1 T cells in endometriosis women compared with healthy donors; however, no differences were found for Vδ2 T cells between endometriosis patients and healthy controls. Interestingly, we described the presence of both Vδ1 and Vδ2 γδ T cell subsets in the peritoneal fluid.

Next, we demonstrated for the first time the cytotoxicity of γδ T-cell subsets against endometrial tumor cell lines including Ishikawa, KLE, and RL95-2. Both Vδ1 and Vδ2 γδ T cells were able to lyse tumor cell lines at low 5:1 E:T ratios with specific lysis ranging between 20% and 68% in the 4-h killing assays. We have shown earlier similar cytotoxicity of γδ T cells against solid tumor cell lines including DU145 (prostate), MCF7 (breast), and A498 (renal) carcinomas (31). Together, our results show frequencies of γδ T-cell subsets in endometriosis patients and their cytotoxicity function against endometrial tumor cell lines. Recent studies have highlighted the correlation of tumor-infiltrating γδ T lymphocytes with patient disease outcome that further confirms the role of γδ T cells in cancer immune surveillance (33, 34). Importantly, γδ T lymphocytes are being intensively investigated towards better clinical applications and new immunotherapeutic interventions (35–37).

In order to elucidate possible mechanisms involved in γδ T-cell cytotoxicity, we chose the EphA2 as it is often overexpressed in many cancers including endometrial carcinomas (28, 29) and also ranked 25th of cancer antigens prioritized for translational research (38). We showed high expression of the EphA2 receptor on KLE and RL-95 endometrial tumor cell lines, and these were used as targets in the EphA2 blocking studies. First, we showed reduced cytotoxicity of Vδ1 T cells after we preincubated KLE and RL-95 target cells with the EphA2 antibody prior to 4-h cytotoxicity assays. Second, we used the EPHA2 small-molecule inhibitor ALW-II-41-27 on KLE endometrial tumor cells and also showed specific lysis significantly reduced at both 5:1 and 10:1 E:T ratios. Third, to confirm the effect of EphA2 inhibition, we generated EPHA2 knockout in endometrial cell line RL95-2 by the CRISPR/Cas9 method and showed significantly reduced specific lysis by Vδ1 T cells in knockout *versus* wild type at 5:1 and 10:1 E:T ratios. Fourth, to further validate the inhibition of cell lysis by tumor-reactive Vδ1 T cells, we used EPHA2 knockouts in renal and colon carcinoma cell lines. Both A-498 KO and HT-29 KO cells showed significant protection from specific lysis mediated by Vδ1 T cells compared with WT cells at 10:1 ratio.

In recent years, studies have been accumulating on differential expression of Eph receptors and their ligands. In particular, the EphA2 triggers cellular events that are deregulated and implicated in carcinogenesis (39). In normal adult tissue, EphA2 expression is absent or present at low levels whereas in malignant cells is overexpressed and functions as a powerful oncoprotein. Targeting Eph receptors with antibodies, peptides and small molecule inhibitors have been widely explored (40–42). Targeting EphA2 is especially attractive in ovarian cancer, in which overexpression is present in over 75% of cases. It was shown in multiple preclinical models of ovarian, breast, and pancreatic cancers that inducing EphA2 downregulation by antibody-mediated inhibition of signaling, antibody-mediated downregulation of total EphA2 expression, and siRNA-mediated inhibition of expression the tumor growth is decreased, further prolongs survival and inhibits angiogenesis (43). Similarly, the pharmacologic inhibition of EPHA2 by the small molecule inhibitor ALW-II-41-27 reduced the viability of resistant tumor cells and inhibited tumor growth *in vivo* in lung cancer models (44). Moreover, high expression of EphA2 was found in endometrial carcinoma and was significantly associated with adverse patient outcome (45).

In summary, we showed for the first time the infiltration of Vδ1 and Vδ2 in peritoneal fluid samples in patients with endometriosis. We determined inherent γδ T-cell cytotoxic reactivity of both subsets from healthy donors against endometrial tumor targets. Importantly, we found that blocking of EphA2 expression significantly inhibits cytotoxicity of tumor reactive Vδ1 γδ T cells. Modifications of EphA2 expression may alter the susceptibility to Vδ1 γδ T-cell-mediated tumor recognition and killing that might be highly relevant in therapies targeting EphA2 in solid tumors and EphA2-positive leukemia (46). Most recent study has identified EphA2 as an antigen recognized by a Vδ1 TCR (30). Our functional data of blocking EphA2 on three different solid tumor cell lines by CRISPR/Cas9 had significantly modified Vδ1 γδ T-cell-mediated tumor lysis. Further expression and functional studies are warranted to demonstrate the therapeutic values of inhibiting the EphA2 in different malignancies, which may however compromise the antitumor Vδ1 γδ T-cell cytotoxicity.

## Data Availability Statement

The raw data supporting the conclusions of this article will be made available by the authors, without undue reservation.

## Ethics Statement

The studies involving human participants were reviewed and approved by Ethics Committee of Faculty of Medicine, Masaryk University. The patients/participants provided their written informed consent to participate in this study.

## Author Contributions

AK designed the study, performed the experiments, analyzed and interpreted data, and wrote the manuscript. MP and BK contributed to research and collected and analyzed the data. IS and RH are in charge of patient accrual and collected the clinical data. RH and MP critically reviewed the manuscript. All authors contributed to the article and approved the submitted version.

## Funding

This study was supported by the Ministry of Health of the Czech Republic (grant number NV19-05-00410 to AK) and the Ministry of Health, Czech Republic-Conceptual Development of Research Organization (FNBr, 65269705 to RH). All rights reserved.

## Conflict of Interest

The authors declare that the research was conducted in the absence of any commercial or financial relationships that could be construed as a potential conflict of interest.

## Publisher’s Note

All claims expressed in this article are solely those of the authors and do not necessarily represent those of their affiliated organizations, or those of the publisher, the editors and the reviewers. Any product that may be evaluated in this article, or claim that may be made by its manufacturer, is not guaranteed or endorsed by the publisher.
